# *α*-Synuclein Attenuates Maneb Neurotoxicity through the Modulation of Redox-Sensitive Transcription Factors

**DOI:** 10.1155/2023/5803323

**Published:** 2023-04-18

**Authors:** M. A. Conde, N. P. Alza, M. I. Funk, A. Maniscalchi, O. N. Benzi Juncos, I. Berge, R. M. Uranga, G. A. Salvador

**Affiliations:** ^1^National Scientific and Technical Research Council-Consejo Nacional de Investigaciones Científicas y Técnicas (CONICET), Instituto de Investigaciones Bioquímicas de Bahía Blanca (INIBIBB), Camino La Carrindanga Km 7, B8000FWB Bahía Blanca, Argentina; ^2^Universidad Nacional del Sur (UNS), Departamento de Biología, Bioquímica y Farmacia, Bahía Blanca, Argentina; ^3^UNS, Departamento de Química, Bahía Blanca, Argentina

## Abstract

The accumulation and aggregation of *α*-synuclein is a pathognomonic sign of Parkinson's disease (PD). Maneb (MB) exposure has also been reported as one environmental triggering factor of this multifactorial neurodegenerative disease. In our laboratory, we have previously reported that mild overexpression of *α*-synuclein (200% increase with respect to endogenous neuronal levels) can confer neuroprotection against several insults. Here, we tested the hypothesis that *α*-synuclein can modulate the neuronal response against MB-induced neurotoxicity. When exposed to MB, cells with endogenous *α*-synuclein expression displayed increased reactive oxygen species (ROS) associated with diminished glutamate-cysteine ligase catalytic subunit (*GCLc*) and hemeoxygenase-1 (*HO-1*) mRNA expressions and upregulation of the nuclear factor erythroid 2-related factor 2 (NRF2) repressor, BTB domain and CNC homolog 1 (BACH1). We found that *α*-synuclein overexpression (wt *α*-syn cells) attenuated MB-induced neuronal damage by reducing oxidative stress. Decreased ROS found in MB-treated wt *α*-syn cells was associated with unaltered *GCLc* and *HO-1* mRNA expressions and decreased BACH1 expression. In addition, the increased SOD2 expression and catalase activity were associated with forkhead box O 3a (FOXO3a) nuclear compartmentalization. Cytoprotective effects observed in wt *α*-syn cells were also associated with the upregulation of silent information regulator 1 (SIRT1). In control cells, MB-treatment downregulated glutathione peroxidase 4 mRNA levels, which was coincident with increased ROS content, lipid peroxidation, and mitochondrial alterations. These deleterious effects were prevented by ferrostatin-1, an inhibitor of ferroptosis, under conditions of endogenous *α*-synuclein expression. The overexpression of *α*-synuclein attenuated MB toxicity by the activation of the same mechanisms as ferrostatin-1. Overall, our findings suggest that mild overexpression of *α*-synuclein attenuates MB-induced neurotoxicity through the modulation of NRF2 and FOXO3a transcription factors and prevents cell death probably by intervening in mechanisms associated with ferroptosis. Thus, we postulate that early stages of *α*-synuclein overexpression could be potentially neuroprotective against MB neurotoxicity.

## 1. Introduction

It has been well-established that changes in *α*-synuclein expression or impairment in its degradation constitute a hallmark of Parkinson's disease (PD). It has also been reported that *SNCA* promoter polymorphisms and epigenetic changes are involved in the pathogenesis of this multifactorial disease [1–3]. Regarding environmental factors, it has been shown that *α*-synuclein expression in neuronal cells can be upregulated by exposure to several pesticides, such as rotenone, paraquat, maneb (MB), and glyphosate [4]. However, little is known about the signaling mechanisms that participate in the pesticide-induced neuronal response when *α*-synuclein is overexpressed.

MB is a widely used fungicide belonging to the family of dithiocarbamate pesticides. Its toxicity has gained attention in neurobiology research due to growing evidence linking occupational and residential human exposure as an environmental factor associated with Parkinsonism [5, 6]. Most of the studies aimed at the understanding of MB toxicity have been carried out over the basis of pesticide coexposure using paraquat. Transcriptome-metabolome-wide association studies showed common interactions in toxicological mechanisms during the combined exposure to both pesticides [7]. *In vivo* experiments have also shown alterations in specific target genes related with PD, such as tyrosine hydroxylase, dopamine transporter, and vesicular monoamine transporter 2 [8]. The loss of motor function due to the apoptosis of dopaminergic neurons in mouse models was also reported as a consequence of the MB-paraquat synergistic effect [9]. In addition, it has been shown that the MB-paraquat combination disrupts mitochondrial function, increasing oxidative stress and reactive oxygen species (ROS) production and contributing to the mislocalization of the forkhead box O transcription factors (FOXO) [10]. Experiments in transgenic mice overexpressing *α*-synuclein and exposed to MB-paraquat combination demonstrated that FOXO impairment was related to adult neurogenesis, thus providing a novel function for these transcription factors in PD [10]. FOXO3 downstream signaling was found to counteract *α*-synuclein accumulation and proteotoxicity, promoting neuronal survival in the *substantia nigra* [11]. In line with these findings, we have previously reported that neuronal FOXO3a signaling participates in cytoprotection in different cellular models of neuronal injury [12, 13].

Another transcription factor involved in the response to MB toxicity in the nervous system is the nuclear factor erythroid 2-related factor 2 (NRF2). Specifically, *NRF2* knockout mice showed hippocampal prooxidant effect when exposed to MB mainly due to an increase in lipid peroxides as a consequence of glutathione peroxidase 4 downregulation [14]. In line with this, resveratrol has been shown to promote NRF2 activation in cellular and mouse models of pesticide-induced Parkinsonism [15]. Divergent mechanisms of neuronal response during paraquat and MB exposure in the downstream NRF2 signaling peroxiredoxin/thioredoxin system have been reported [16]. So far, much of the evidence about cellular mechanisms triggered during pesticide neurotoxicity comes from a combination of them, and few studies are available reporting the effect of MB alone. In consonance, groundbreaking findings from metabolomic approaches have demonstrated alterations in central carbon metabolism and thiol redox status caused by the exposure to MB as a unique injury stimulus in neuronal cell cultures [17]. In addition, MB exposure has been associated with a recently described cell death mechanism, named as ferroptosis [18].

The above-described evidence points to not yet clearly elucidated but specific mechanisms triggered by MB toxicity involving transcriptional regulation of cellular responses against injury and *α*-synuclein biology. However, most of the studies were made in animal models where the interaction of multiple cell types contributes to the complexity of dissecting specific mechanisms leading to neuronal injury. Considering this and the fact that MB exerts its action by crossing the blood-brain barrier, the use of cell culture constitutes an appropriate model for elucidating the molecular pathways involved in neurotoxicity events [19, 20]. Consequently, the aim of our study was to describe the effect of MB exposure in neuronal cells with endogenous and overexpressed levels of *α*-synuclein. Our study was mainly focused on the neuronal response regarding FOXO3a and NRF2 signaling and the final cellular outcome during MB exposure.

## 2. Materials and Methods

### 2.1. Materials

#### 2.1.1. Antibodies

Rabbit polyclonal anti-*α*-synuclein (C-20-R) (catalog no. sc-7011-R), rabbit polyclonal anti-phospho-*α*-synuclein (catalog no. sc-135638), mouse monoclonal anti-*β*-actin (catalog no. sc-47778), mouse monoclonal IgG2A hnRNP (catalog no. sc-32301), mouse monoclonal anti-NRF2 (catalog no. sc-365949), mouse monoclonal anti-SOD2 (catalog no. sc-137254), mouse monoclonal anti-BACH1 (catalog no. sc-271211), mouse monoclonal anti-SIRT1 (catalog no. sc-74465), polyclonal horseradish peroxidase- (HRP-) conjugated mouse anti-rabbit IgG (catalog no. sc-2357), and normal anti-mouse IgG2A (catalog no. sc-3878) were purchased from Santa Cruz Biotechnology, Inc. (Santa Cruz, CA, USA). Rabbit polyclonal anti-phosphoSer473-AKT (catalog no. cs-9271), rabbit polyclonal anti-AKT (catalog no. cs-9272), and rabbit polyclonal anti-FOXO3a (catalog no. cs-2497) were purchased from Cell Signaling Technology (Beverly, MA, USA). Mouse monoclonal anti-*α*-tubulin (catalog no. NB-690) was purchased from Novus Biologicals LLC (Centennial, CO, USA). HRP-conjugated sheep anti-mouse IgG (catalog no. NA931V) was obtained from Amersham Biosciences (Chicago, USA). Alexa Fluor 488 goat anti-mouse (catalog no. A-11001), polyclonal Alexa Fluor 488-conjugated goat anti-rabbit (catalog no. A-11034), polyclonal Alexa Fluor 594-conjugated goat anti-mouse (catalog no. A-11032), and Alexa Fluor 546-conjugated goat anti-rabbit (catalog no. A-11035) were purchased from Thermo Fisher-Invitrogen (CABA, Argentina).

#### 2.1.2. Reagents

2′,7′-Dichlorofluorescein diacetate (catalog no. D399) and MitoTracker Red CMXRos (catalog no. M7512) were obtained from Molecular Probes (Eugene, OR, USA). Polyvinylidene difluoride (PVDF) membranes were purchased from EMD Millipore (Bedford, MA, USA). Dulbecco's Modified Eagle Medium (DMEM) (catalog no. 52100047), trypsin (catalog no. 15090046), and antibiotic-antimycotic (catalog no. 15240062) were obtained from Gibco (CABA, Argentina). Geneticin (G418) (catalog no. sc-29065) and bovine serum albumin (BSA, catalog no. sc-2323) were purchased from Santa Cruz Biotechnology, Inc. Fetal bovine serum (FBS) was obtained from Internegocios (Mercedes, Buenos Aires, Argentina). Dimethyl sulfoxide (catalog no. D2650), 2-(4-morpholinyl)-8-phenyl-1(4H)-benzopyran-4-one hydrochloride (LY294002; catalog no. L9908), 3-(4,5-dimethylthiazol-2-yl)-2,5-diphenyltetrazolium bromide (MTT; catalog no. M2128), ferrostatin-1 (Fer-1; catalog no. SML0583), and Triton™ X-100 (catalog no. T9284) were purchased from Sigma-Aldrich Co. (St. Louis, MO, USA). Lipofectamine 2000 (catalog no. 11668019), TRIzol reagent (catalog no. 15596-026), and Hoechst (catalog no. H-1399) were purchased from Thermo Fisher-Invitrogen (CABA, Argentina). The kit (LDH-P UV AA) for measuring lactate dehydrogenase (LDH) activity was generously supplied by the Wiener Laboratory (Rosario, Santa Fe, Argentina; catalog no. 1521303). PCR primers for human *GAPDH*, *GCLc*, and *HO-1* were synthetized by GEN Biotech (CABA, Argentina). Luna Universal Master Mix (catalog no. M3003S) was from New England BioLabs-Migliore Laclaustra (CABA, Argentina). Trichostatin A (TSA, Sigma, catalog no. T8552) was kindly provided by Dr. Natalia De Miguel (Instituto Tecnológico de Chascomus, INTECH, Argentina). All other chemicals used in the present study were of the highest purity available.

### 2.2. Cell Culture

The human neuroblastoma cell line IMR-32 was obtained from ATCC. Cells were transfected with the expression vector containing human wild-type *α*-synuclein or the empty vector pcDNA using Lipofectamine 2000 following the manufacturer's protocol. After 72 h posttransfection, cells were treated with 400 *μ*g/mL G418 for selection and grown in DMEM high-glucose medium supplemented with 200 *μ*g/mL G418, 10% (*v*/*v*) FBS, 100 U/mL penicillin, 100 *μ*g/mL streptomycin, and 0.25 *μ*g/mL amphotericin B in a humidified atmosphere of 5% CO_2_ at 37°C. Western blot and immunocytochemistry studies were used to monitor *α*-synuclein expression levels. Cells were negative for *Mycoplasma* sp.

### 2.3. Experimental Treatments

Cells were grown to 80-90% confluence and treated with 24 *μ*M MB or vehicle (dimethyl sulfoxide, 0.15%) for 48 h unless otherwise specified. Treatments with inhibitors were performed as follows: after replacement of DMEM with serum-free medium, 10 *μ*M LY294002, 200 nM TSA, and 6 *μ*M Fer-1 were coincubated with MB for 48 h. Controls were treated with the vehicle alone.

### 2.4. Cell Viability

Cell viability was assessed by the MTT reduction assay. Metabolically, viable cells reduced the MTT, a water-soluble tetrazolium salt, to a colored, water-insoluble formazan salt. After treatments, cells seeded in 96-well plates (1 × 10^4^ cells/well) were incubated for 2 h at 37°C in serum-free medium containing 0.5 mg/mL MTT. Subsequently, the medium was removed, and the formazan crystals were dissolved with 20% (*w*/*v*) sodium dodecyl sulfate, pH 4.6. The reaction was measured spectrophotometrically at 570 nm [12]. Results are expressed as a percentage of the control condition.

### 2.5. Cell Oxidant Levels

Cellular oxidant levels were evaluated using the probe 5 (or 6)-carboxy-2′7′-dichlorodihydrofluorescein diacetate (H2DCFDA), which is converted to a fluorescent compound after oxidation. Following treatments, the medium was replaced by DMEM containing 10 *μ*M H2DCFDA and incubated at 37°C for 30 min. After the medium was removed, cells were rinsed three times with phosphate buffer saline (PBS) and then lysed in a buffer containing PBS and 0.1% NP-40. The fluorescence was measured at *λ*ex = 495 nm and *λ*em = 530 nm in a Fluoroskan Ascent FL microplate fluorometer [12] Results are expressed as arbitrary units normalized to the protein content.

### 2.6. LDH Release

After treatments, the incubation medium was centrifuged at 1000 × *g* for 10 min at 4°C. The supernatant was used to determine LDH leakage by measuring at 340 nm with the LDH-P UV AA kit, following the manufacturer's instructions [13].

### 2.7. Determination of Lipid Peroxidation

Lipid peroxidation was measured by the thiobarbituric acid reactive substance (TBARS) assay [12]. Briefly, after treatments, 0.5 mL of 30% trichloroacetic acid was added to 0.25 mL of cellular homogenates. After the addition of 50 *μ*L of 5N HCl and 500 *μ*L of 0.75% thiobarbituric acid, tubes were capped and the mixtures were heated at 100°C for 15 min in a boiling water bath. Samples were centrifuged at 1000 × *g* for 10 min, and supernatants were used to determine TBARS spectrophotometrically at 535 nm. Results are expressed as arbitrary units normalized to the protein content.

### 2.8. Catalase Activity

In whole cellular lysates, catalase activity was measured following the procedure of Aebi [21]. After lysates were obtained with 50 mM phosphate buffer, the activity was calculated using the rate of degradation of the enzyme substrate, hydrogen peroxide, at 240 nm. The enzyme activity was expressed as the rate constant of a first-order reaction normalized to the protein content.

### 2.9. Western Blot

Cells were seeded in 60 mm dishes and, after treatments, rinsed with PBS, scraped, and centrifuged. The supernatant was discarded, and the pellet was homogenized with 60-80 *μ*L of a lysis buffer (50 mM Tris pH 7.5, 150 mM NaCl, 0.1% Triton X-100, 1% NP-40, 2 mM EDTA, 2 mM EGTA, 50 mM NaF, 2 mM *β*-glycerophosphate, 1 mM Na_3_VO_4_, 10 *μ*g/mL leupeptin, 5 *μ*g/mL aprotinin, 1 *μ*g/mL pepstatin, 0.5 mM PMSF, and 0.5 mM DTT). The homogenized samples were kept on ice for 60 min, vortexing every 5 min, and then centrifuged at 15000 × *g* for 15 min. The protein content was measured in aliquots from the supernatants by the Bradford method [22]. Cell lysates were mixed with the Laemmli buffer 4x, and samples with 25-50 *μ*g of protein were separated by reducing 10% polyacrylamide gel electrophoresis and transferred into PVDF membranes. Molecular weight standards were run in the same gel.

After blockage with 5% (*w*/*v*) nonfat dry milk in TBS-T buffer (20 mM Tris–HCl (pH 7.4), 100 mM NaCl, and 0.1% (*w*/*v*) Tween 20) for 1 h at room temperature, membranes were washed three times with TBS-T buffer and incubated with primary antibodies overnight at 4°C. Then, membranes were washed three times with TBS-T buffer and incubated with the corresponding HRP-conjugated secondary antibody for 2 h at room temperature. Finally, membranes were again washed three times with TBS-T buffer, and enhanced chemiluminescence with X-ray films was used for the detection of immunoreactive bands. The quantification of immunoreactive bands was performed using FIJI platform [23].

### 2.10. Cytoplasmic and Nuclear Fractionation

Nuclear and cytosolic fractions were isolated as described previously [13, 24, 25]. Briefly, after treatments, the medium was discarded, and cells were rinsed with PBS, scraped, and centrifuged at 800 × *g* for 8 min. The pellet was resuspended in 40 *μ*L of buffer A (10 mM HEPES (pH 7.9), 1.5 mM MgCl_2_, 10 mM KCl, 0.5 mM DTT, 0.1% NP-40, 2 *μ*g/*μ*L leupeptin, 1 *μ*g/*μ*L aprotinin, and 1 *μ*g/*μ*L pepstatin). After incubating for 10 min on ice, cell suspension was centrifuged for 2 min at 12000 × *g* at 4°C. The supernatant containing the cytosolic fraction was extracted, and the pellet was then resuspended in 40 *μ*L of buffer B (10 mM HEPES (pH 7.9), 1.5 mM MgCl_2_, 420 mM NaCl, 0.5 mM DTT, 0.2 mM EDTA, 25% glycerol, 0.5 mM PMSF, 2 *μ*g/mL leupeptin, 1 *μ*g/mL aprotinin, and 1 *μ*g/mL pepstatin), subjected again to lysis for 20 min on ice and centrifugation at 10000 × *g* for 15 min at 4°C. The supernatant contained the nuclear fraction. Proteins were quantified by the Bradford method. Samples were stored at -20°C until used for Western blot analyses.

### 2.11. Immunofluorescence Microscopy

Cells were grown onto glass coverslips. After treatments, cells were fixed with 4% paraformaldehyde in PBS for 20 min. For the immunostaining, cells were permeabilized and blocked with 2% BSA in PBS and 0.1% Triton X-100 for 45 min at room temperature. Next, cells were incubated with the appropriate primary antibody (1 : 100 in PBS, 2% BSA, and 0.1% Triton X-100) for 1 h at room temperature. After washing three times with PBS, cells were incubated with the corresponding fluorescent secondary antibody for 1 h (1 : 300 in PBS and 2% BSA) and Hoechst for nuclear staining for 7 min. Then, coverslips were mounted, and analysis was performed with a Nikon Eclipse E600 microscope or a confocal laser scanning microscope Leica DMIRE2. The fluorescence intensity was quantified using the platform FIJI [23], and at least 100 cells for each condition were analyzed from three independent cell cultures.

### 2.12. Quantitative RT-PCR (RT-qPCR)

Total RNA samples were prepared using TRIzol reagent following the manufacturer's protocol and resuspended in nuclease-free water. Concentration and purity of isolated RNA were determined by a PicoDrop Pico100 spectrophotometer. Samples were stored at −80°C until use. To synthesize cDNA with the High-Capacity RNA-to-cDNA™ Kit (Applied Biosystems), aliquots containing 1 *μ*g total RNA were used. qRT-PCR was performed in a final volume of 15 *μ*L using Luna Universal Master Mix and 0.2 *μ*M of each primer. Rotor-Gene 6000 was used to determine gene expression levels. Ct values from 3 different experiments were normalized according to the 2^−*ΔΔ*Ct^ method using *GAPDH* as reference gene. Gene-specific primer sequences designed for qPCR were to *GAPDH*, forward: TTCACCACCATGGAGAAGGC and reverse: AGTGATGGCATGGACTGTGGTC; *GCLc*, forward: GGAAGTGGATGTGGACACCAGA and reverse: GCTTGTAGTCAGGATGGTTTGCG; *HO-1*, forward: CCAGGCAGAGAATGCTGAGTTC and reverse: AAGACTGGGCTCTCCTTGTTGC; and *GPX4*, forward: ACAAGAACGGCTGCGTGGTGAA and reverse: GCCACACACTTGTGGAGCTAGA.

### 2.13. Glutathione (GSH) Cellular Content

GSH was measured in total cellular extracts by using a modification of a previously reported colorimetric 5,5′-dithiobis(2-nitrobenzoic acid)- (DTNB-) based method [12]. Briefly, samples used for GSH determinations were incubated in the absence or in the presence of N-ethylmaleimide. The reaction between GSH and DTNB was measured at 412 nm.

### 2.14. Evaluation of Mitochondrial Alterations

Staining with 200 nM MitoTracker Red CMXRos for 30 min at 37°C was used to assess mitochondrial membrane potential. Fluorescence was visualized with a Nikon Eclipse E600 microscope. Images were analyzed for fluorescence quantification using the parameter “RawIntDen” of Fiji software which is the pixel sum of selected areas [26]. This data was calculated in 60 cells in three independent experiments. To analyze MitoTracker Red diffusion as a marker of defects of mitochondrial membrane potential, profile plots were created with ImageJ software [27].

### 2.15. Statistical Analysis

The data were summarized as means ± SD of three independent experiments and analyzed using a specific software (GraphPad 8.0, free trial). The variance similarity was determined by the *F*-test. Statistical significance was determined by one-way ANOVA followed by Tukey's test. *p* values lower than 0.05 were considered statistically significant; ^∗^, ^∗∗^, or ^∗∗∗^ represent *p* < 0.05, *p* < 0.01, and *p* < 0.001, respectively.

## 3. Results

### 3.1. Differential Effect of MB on *α*-Synuclein Expression and Phosphorylation in Control and wt *α*-syn Cells

**Figure 1:**
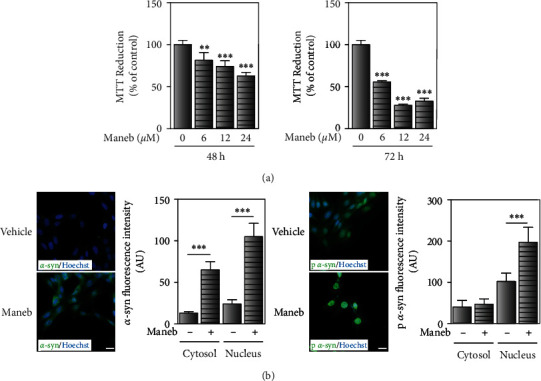
Effect of MB exposure on the expression and phosphorylation of *α*-synuclein. IMR-32-neuroblastoma cells were exposed to MB or its vehicle (dimethyl sulfoxide) to characterize the response to pesticide exposure. (a) Cellular viability was measured by the MTT reduction assay in cells exposed to MB 6, 12, and 24 *μ*M for 48 h (left) and 72 h (right) or vehicle. Results are expressed as a percentage of the control and represent mean ± SD (*n* = 3–4). (b) Immunocytochemistry studies showing *α*-synuclein (*α*-syn) expression (left) and phosphorylation (right) in IMR-32 cells exposed to MB (24 *μ*M) or vehicle for 48 h. Hoechst was used as nuclear marker. The fluorescence intensity was quantified in cytosol and nucleus using ImageJ, and values are expressed as arbitrary units. Scale bar: 20 *μ*m. (a, b) ^∗∗^*p* < 0.01 and ^∗∗∗^*p* < 0.001 for each condition with respect to the corresponding control; one-way ANOVA and Tukey's post hoc test.

MB is a fungicide responsible for causing PD symptoms [28]. The fact that pesticide exposure is a well-known environmental risk factor for Parkinsonism prompted us to study the neuronal response under MB toxicity and the involvement of *α*-synuclein in this paradigm. In order to assess the pesticide cytotoxicity, the MTT reduction assay was performed at a range of MB concentrations (6, 12, and 24 *μ*M) and exposure times (48 and 72 h). Our results revealed concentration-dependent cytotoxicity of MB at 48 h (Figure 1(a)). Even though the pesticide was more toxic at 72 h, its effect was independent of the concentration at this exposure time. Due to the moderate toxicity (about 40% of cell viability reduction), 24 *μ*M MB for 48 h was selected as the cell damage condition for subsequent experiments. Taking into account that not only *α*-synuclein accumulation but also its phosphorylation at S129 are considered pathological signs, we evaluated the effect of MB on the protein expression and phosphorylation [29]. Our experiments showed that MB enhanced the levels of both *α*-synuclein and its phosphorylated form in IMR-32 cells (Figure 1(b)).

**Figure 2:**
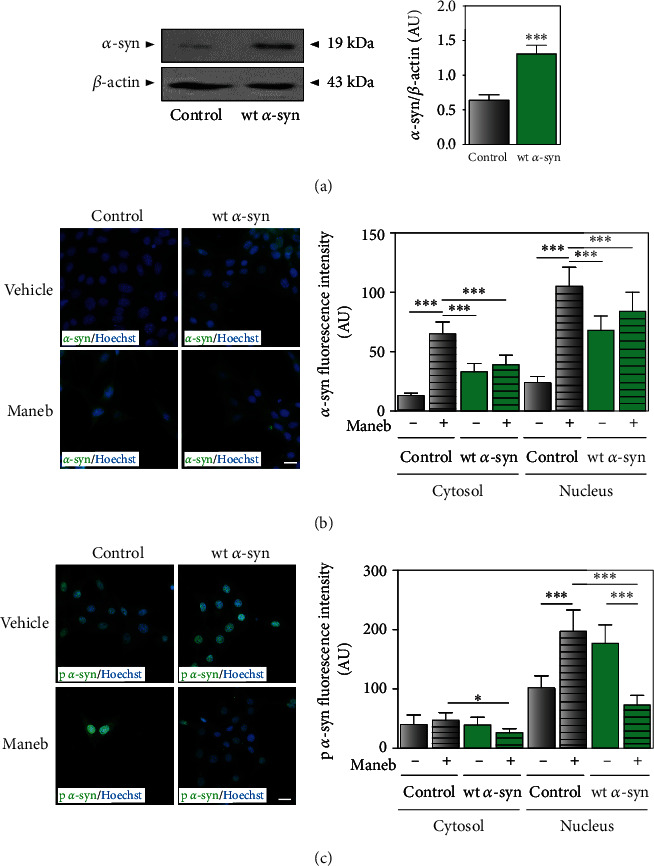
*α*-Synuclein expression and phosphorylation are affected to a different extent by MB in cells with endogenous and overexpressed levels of the protein. (a) *α*-Synuclein expression levels were determined in wt *α*-syn and control cells (IMR-32 cells stably transfected with pcDNA-human wild-type *α*-synuclein plasmid or the empty vector, respectively) by Western blot in total cellular lysates. *β*-Actin was used as loading control. A representative blot of three different experiments is shown. Band quantification was performed with ImageJ. Results are expressed as arbitrary units and represent mean ± SD (*n* = 3). (b, c) Analysis of *α*-synuclein expression and phosphorylation in control and wt *α*-syn cells exposed to MB (24 *μ*M) or vehicle for 48 h was performed by immunocytochemistry studies. Hoechst was used as nuclear marker. Fluorescence intensity quantification was performed with ImageJ, and results are expressed as arbitrary units. Scale bars: 20 *μ*m. (a–c) ^∗^*p* < 0.05 and ^∗∗∗^*p* < 0.001 for each condition with respect to the corresponding control; one-way ANOVA and Tukey's post hoc test.

To further characterize how *α*-synuclein expression was altered by MB exposure, our laboratory established a cellular model using IMR-32 cells stably transfected with the human wild-type *α*-synuclein-pcDNA3 vector (wt *α*-syn cells and *α*-synuclein overexpression) or the empty vector (control cells and endogenous *α*-synuclein expression). As Western blot shows in Figure 2(a), *α*-synuclein expression was increased 2-3 times in wt *α*-syn cells compared to controls. While a rise in *α*-synuclein levels was detected in control cells exposed to MB, to our surprise, expression changes were not observed in wt *α*-syn cells challenged with the pesticide (Figure 2(b)). Regarding *α*-synuclein phosphorylation, it was enhanced by MB treatment in controls (Figure 2(c)), which was in agreement with the findings in nontransfected cells. Controversially, the levels of *α*-synuclein phosphorylated were reduced after MB exposure in wt *α*-syn neurons, as shown in Figure 2(c).

### 3.2. *α*-Synuclein Attenuates MB-Induced Oxidative Stress

**Figure 3:**
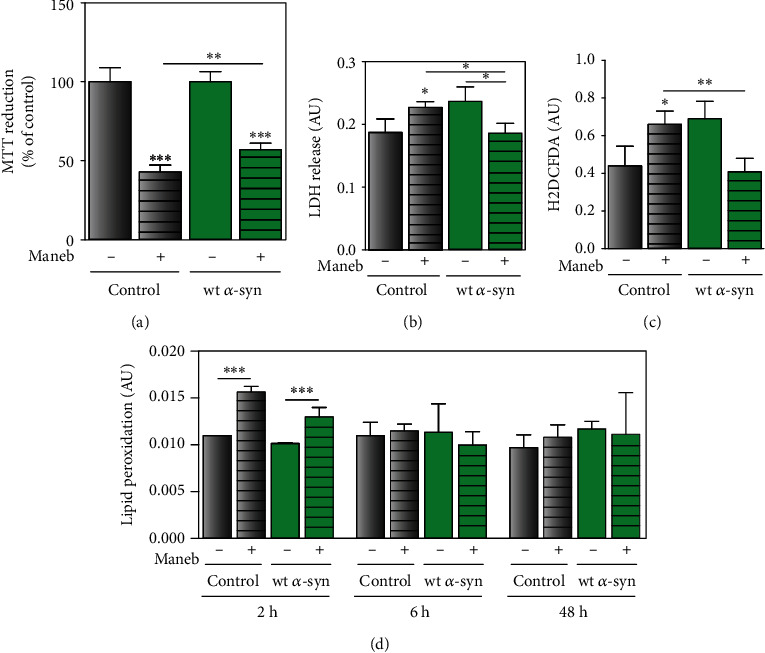
MB-induced injury is attenuated by *α*-synuclein overexpression. Cell injury and redox markers were evaluated under endogenous expression and overexpression of *α*-synuclein (control and wt *α*-syn cells, respectively) after exposure to MB (24 *μ*M) or vehicle for 48 h. (a) Cellular viability was measured by the MTT reduction assay. (b) Plasma membrane integrity was characterized through LDH leakage. (c) Cellular oxidant levels were determined using the probe H2DCFDA. (d) Lipid peroxidation was determined by TBARS assay at different MB time exposures. (a–d) Results are expressed as arbitrary units or percentage of the control and represent mean ± SD (*n* = 3–4). ^∗^*p* < 0.05, ^∗∗^*p* < 0.01, and ^∗∗∗^*p* < 0.001 for each condition with respect to the corresponding control; one-way ANOVA and Tukey's post hoc test.

Based on the above-mentioned data, we hypothesized that MB toxicity is counteracted when *α*-synuclein is overexpressed. To test our hypothesis, cellular viability was analyzed after MB exposure in cells with endogenous levels and overexpression of *α*-synuclein. Our results showed that wt *α*-syn cells were less sensitive to MB than control cells (Figure 3(a)), pointing towards a protective role of *α*-synuclein against the pesticide toxicity. To reinforce these data, membrane permeability was evaluated through the LDH leakage assay. In control cells, LDH release was augmented upon MB treatment; however, LDH leakage was diminished in cells overexpressing *α*-synuclein challenged with MB (Figure 3(b)).

Given that oxidative stress is considered a key player in MB-induced cellular damage, we also determined ROS production in wt *α*-syn and control cells. As expected, MB caused a 60% increase in ROS in control cells with endogenous levels of *α*-synuclein (Figure 3(c)). Nevertheless, the generation of ROS was reduced after the pesticide exposure in wt *α*-syn cells (40% of reduction) (Figure 3(c)). Furthermore, we observed that MB treatment induced lipid peroxidation to a different extent depending on *α*-synuclein expression levels, being lipid peroxide content 50% and 30% higher in control and wt *α*-syn neurons, respectively (Figure 3(d)). Therefore, these results suggested that middle *α*-synuclein overexpression protects against oxidative stress induced by the pesticide.

### 3.3. *α*-Synuclein Overexpression Differentially Modulates the Transcription Factors NRF2 and FOXO3a

**Figure 4:**
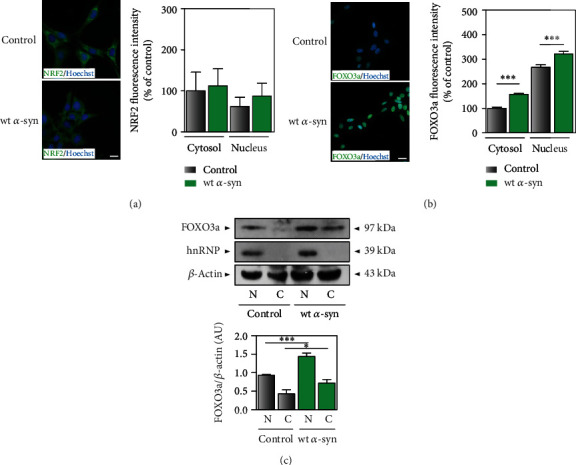
Effect of *α*-synuclein overexpression on the state of transcription factors involved in cellular responses to oxidative stress. (a, b) Immunocytochemistry was used to analyze NRF2 and FOXO3a cellular localization in cells with endogenous and overexpressed levels of *α*-synuclein (control and wt *α*-syn cells, respectively). Hoechst was used as nuclear marker. Fluorescence intensity was quantified using ImageJ. Pictures represent at least three different experiments. Scale bars: 20 *μ*m. (c) Western blot analysis of FOXO3a levels was performed in nuclear (N) and cytosolic (C) fractions. hnRNP was used as nuclear marker and *β*-actin as loading control. Representative blots of three different experiments are shown. Band quantification was performed with ImageJ. (a–c) Results are expressed as percentage of the control or arbitrary units and represent mean ± SD (*n* = 3). ^∗^*p* < 0.05 and ^∗∗∗^*p* < 0.001 for each condition with respect to the control; one-way ANOVA and Tukey's post hoc test.

Our lab and others have deeply investigated how different insults related to neuronal damage, such as the exposure to iron and oligomeric amyloid *β* peptide, trigger signaling pathways implicated in the determination of cellular fate [12, 13, 25]. Given that our results showed that *α*-synuclein prevents MB toxicity, we explored whether the protein modulates crucial pathways involved in antioxidant response. On the one hand, the expression of the cytoprotective transcription factor NRF2 was not affected by *α*-synuclein overexpression, as shown in Figure 4(a). Additionally, NRF2 cellular localization remained unaltered in wt *α*-syn cells, being mainly cytosolic during both endogenous expression and overexpression of *α*-synuclein (Figure 4(a)). On the other hand, the levels of FOXO3a, a transcription factor activated under cellular stress conditions, were augmented by *α*-synuclein overexpression both in nuclear and cytosolic fractions (50% increase) (Figures 4(b) and 4(c)).

### 3.4. The Modulation of NRF2 Downstream Genes Is Divergent under Endogenous Expression and Overexpression of *α*-Synuclein

**Figure 5:**
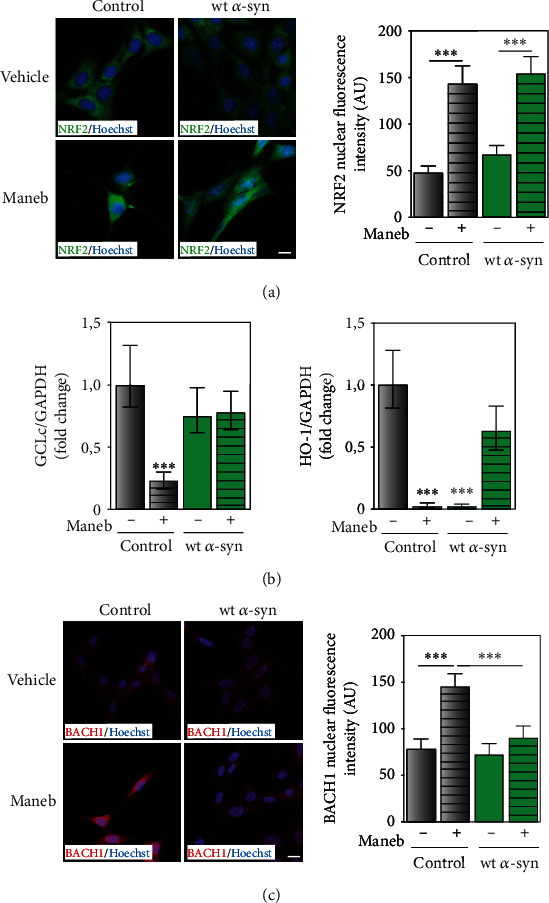
NFR2 pathway is downregulated in cells with endogenous *α*-synuclein upon MB exposure. (a, c) Immunocytochemistry analysis reveals NRF2 and BACH1 nuclear expression in control and wt *α*-syn cells exposed to the pesticide. Hoechst was used as nuclear marker. Fluorescence intensity quantification was assessed with ImageJ. Results are expressed as arbitrary units. The pictures shown are representative of at least three different experiments. Scale bars: 20 *μ*m. (b) mRNA levels of GCLc and HO-1, both NRF2 downstream genes, were determined in cells with endogenous and overexpressed *α*-synuclein in the presence of MB. mRNA expression was assessed by qRT-PCR. Results are expressed as fold change compared to the control and represent mean ± SD (*n* = 3). (a–c) ^∗∗∗^*p* < 0.001 for each condition with respect to the control; one-way ANOVA and Tukey's post hoc test.

Next, we investigated NRF2 cellular localization and downstream gene expression in control and wt *α*-syn cells during MB exposure. Previous studies demonstrated the involvement of NRF2 in cellular protection against MB toxicity [14, 15]. Our results clearly show that MB triggered NRF2 nuclear translocation under endogenous and overexpressed levels of *α*-synuclein (Figure 5(a)). Furthermore, mRNA levels of the NRF2-regulated gene glutamate-cysteine ligase catalytic subunit (*GCLc*) and hemeoxygenase-1 (*HO-1*) were determined through RT-qPCR. Although *GCLc* mRNA levels were not altered by MB in cells overexpressing *α*-synuclein, they were diminished by MB exposure in control cells (80% reduction), as shown in Figure 5(b). Regarding *HO-1* expression, mRNA levels were diminished in control cells exposed to MB, whereas they were restored in wt *α*-syn cells treated with the pesticide (Figure 5(b)). Interestingly and to reinforce our findings, the nuclear localization of the NRF2 transcription repressor BTB domain and CNC homolog 1 (BACH1) was increased upon MB treatment in controls, but it remained unchanged in wt *α*-syn neurons exposed to the pesticide (Figure 5(c)). Thus, our results suggest that, under endogenous *α*-synuclein expression, BACH1 might be responsible for the reduced antioxidant response during MB toxicity.

### 3.5. FOXO3a and SIRT1 Are Involved in *α*-Synuclein Protective Role against MB Toxicity

**Figure 6:**
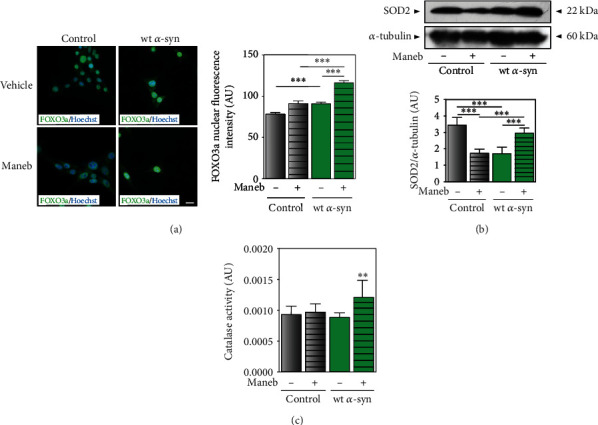
*α*-Synuclein modulates FOXO3a pathway leading to SOD2 upregulation in wt *α*-syn cells after MB treatment. (a) FOXO3a expression and cellular localization were evaluated by immunocytochemistry studies in cells with endogenous levels and overexpressing *α*-synuclein (control and wt *α*-syn cells, respectively) exposed to the pesticide (24 *μ*M) or vehicle for 48 h. Hoechst was used as nuclear marker. Results are expressed as arbitrary units, and ImageJ was used for fluorescence intensity quantification. Scale bar: 20 *μ*m. (b) SOD2 expression in control and wt *α*-syn cells was analyzed by Western blot in total fractions after MB exposure. *α*-Tubulin was used as loading control. One representative blot of three different experiments is shown. Bands of proteins were quantified using ImageJ. Results are expressed as arbitrary units. (c) Catalase activity was measured in control and wt *α*-syn cells after MB exposure. Results are expressed as arbitrary units per mg of protein. (a–c) ^∗∗^*p* < 0.01, and ^∗∗∗^*p* < 0.001 with respect to the corresponding control; one-way ANOVA and Tukey's post hoc test. Results represent mean.

It has been proposed that FOXO3a plays a key role in the response to MB and paraquat coexposure in transgenic mice with *α*-synuclein overexpression that finally impacts neurogenesis [10]. Here, we observed that FOXO3a did not change its subcellular localization in control cells in the presence of MB, whereas its nuclear localization was found to be higher when *α*-synuclein was overexpressed (Figure 6(a)). To understand the role of FOXO3a under MB toxicity, two downstream genes were evaluated, manganese superoxide dismutase (*SOD2*) and catalase. We found that SOD2 protein levels and catalase activity were upregulated in cells overexpressing *α*-synuclein exposed to MB (Figures 6(b) and 6(c)). In agreement and consistently with the injury observed in control cells, under conditions of endogenous *α*-synuclein expression, SOD2 expression diminished and catalase activity remained unchanged after MB treatment (Figures 6(b) and 6(c)). These results argue in favor of the participation of FOXO3a as a modulator of cellular antioxidant defenses.

**Figure 7:**
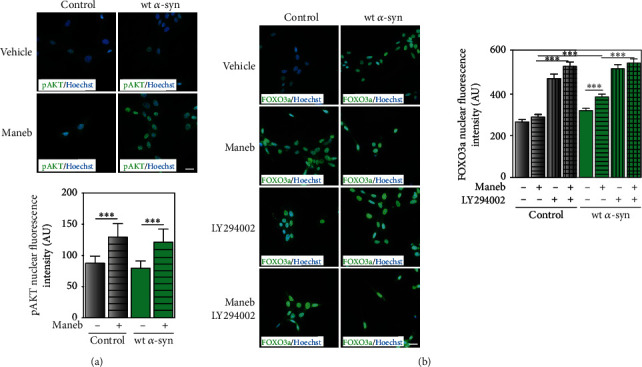
Effect of MB exposure on FOXO3a phosphorylation by the PI3K-AKT pathway in control and wt *α*-syn cells. (a) Immunocytochemistry studies show the expression of phosphorylated AKT in control and wt *α*-syn cells exposed to MB. (b) Nuclear FOXO3a levels were determined by immunocytochemistry under the inhibition of PI3K/AKT pathway using 10 *μ*M LY294002 in the presence of MB. (a, b) Hoechst was used as nuclear marker. Fluorescence intensity was quantified using ImageJ. Results are expressed as arbitrary units. ^∗∗∗^*p* < 0.001 for each condition with respect to the control; one-way ANOVA and Tukey's post hoc test. Scale bars: 20 *μ*m.

The inactivation of FOXO3a by phosphorylation that results in its cytoplasmic accumulation is a mechanism mainly mediated by PI3K/AKT signaling pathway. Thus, the dependence of FOXO3a expression on AKT under MB toxicity was evaluated in control and wt *α*-syn neurons. Immunocytochemistry studies shown in Figure 7(a) demonstrate that MB induced AKT phosphorylation, necessary for FOXO3a inactivation. However, *α*-synuclein overexpression did not affect AKT phosphorylation (Figure 7(a)). Furthermore, when exploring whether AKT was involved in the transcription factor regulation, our findings demonstrated that the blockage of PI3K by LY294002, and subsequent inhibition of AKT phosphorylation, did not change the state of FOXO3a under MB exposure neither in control nor in wt *α*-syn neurons (Figure 7(b)). Consequently, our data suggest that phosphorylation was not a posttranslational modification involved in the regulation of FOXO3a transcriptional activity during any of these experimental scenarios.

**Figure 8:**
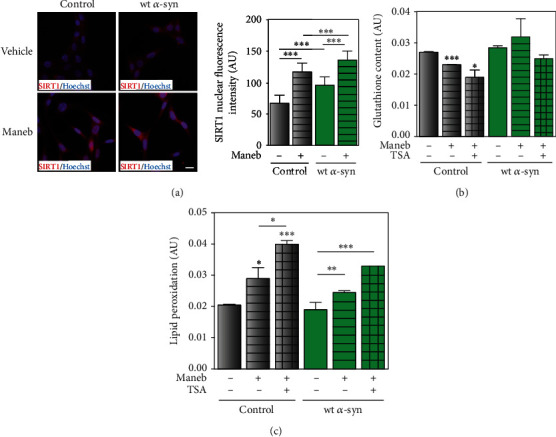
SIRT1 is involved in the antioxidant response in cells overexpressing *α*-synuclein upon MB injury. (a) SIRT1 localization and expression were assessed by immunocytochemistry studies in control and wt *α*-syn cells upon MB treatment. (b, c) After TSA (200 nM) treatment, GSH synthesis and lipid peroxidation were determined in cells with endogenous expression and overexpressing *α*-synuclein exposed to the pesticide. (a–c) Results are expressed as arbitrary units and represent mean ± SD (*n* = 3-4). (a–c) ^∗^*p* < 0.05, ^∗∗^*p* < 0.01, and ^∗∗∗^*p* < 0.001 for each condition with respect to the control; one-way ANOVA and Tukey's post hoc test.

Another intriguing characteristic of wt *α*-syn cells was the increased expression of silent information regulator 1 (SIRT1). This deacetylase has been involved in the regulation of antioxidant defenses and redox signaling [30]. As shown in Figure 8(a), SIRT1 nuclear localization was promoted by *α*-synuclein overexpression as well as MB exposure. To understand the role of SIRT1 in the neuronal response to MB toxicity, we performed experiments in the presence of the deacetylase inhibitor TSA. We observed that MB treatment diminished GSH levels in control cells. After SIRT1 downregulation with TSA, GSH levels were only reduced in control cells exposed to MB as shown in Figure 8(b). Regarding the effect of TSA on oxidative stress after MB exposure, we observed that lipid peroxidation was enhanced in cells with endogenous *α*-synuclein expression (Figure 8(c)). These results suggest that the upregulation of SIRT1 in wt *α*-syn cells participates in the attenuation of MB-induced oxidative injury.

### 3.6. *α*-Synuclein Overexpression Intervenes in MB-Induced Ferroptosis

**Figure 9:**
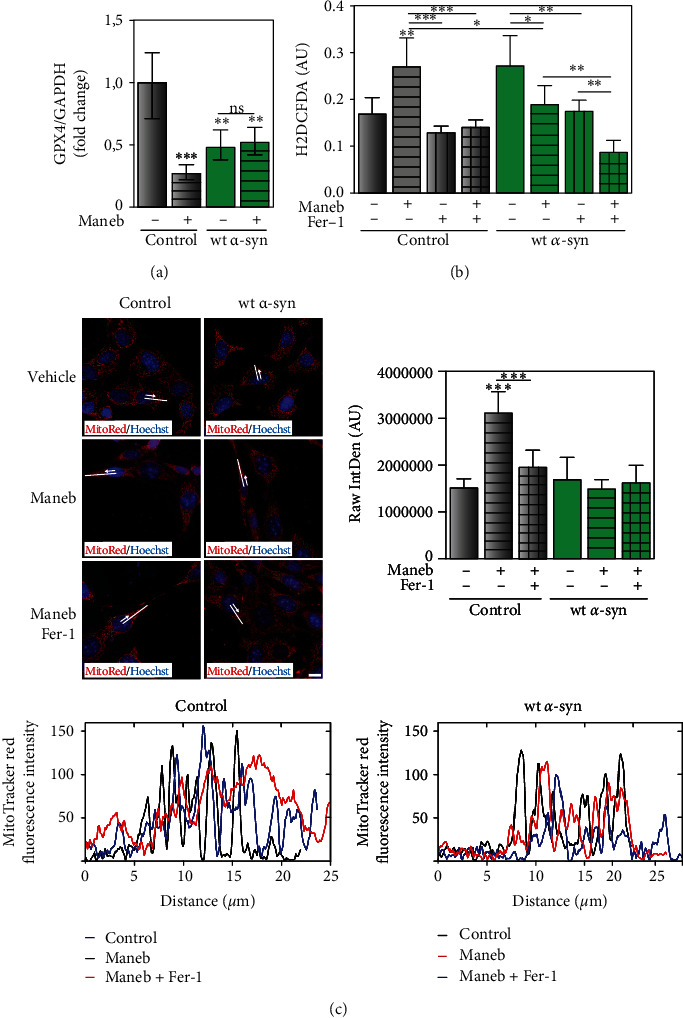
Overexpression of *α*-synuclein attenuates ferroptosis markers triggered by MB exposure. (a) mRNA levels of *GPx4* was determined in cells with endogenous and overexpressed *α*-synuclein in the presence of MB. Results are expressed as fold change compared to the control and represent mean ± SD (*n* = 3). (b) Cellular oxidant levels were determined using the probe H2DCFDA after Fer-1 (6 *μ*M) treatment. Results are expressed as arbitrary units and represent mean ± SD (*n* = 3). (c) Mitochondrial alterations were evaluated using MitoTracker Red staining. Quantification of dye diffusion was determined by the RawIntDen parameter (right) and profile plots (along a line drawn from the middle of the nuclei to the edge of the cell) (down). Results are expressed as arbitrary units and represent mean ± SD (*n* = 3). Scale bar: 20 *μ*m. (a–c) ^∗^*p* < 0.05, ^∗∗^*p* < 0.01, and ^∗∗∗^*p* < 0.001 for each condition with respect to the control; one-way ANOVA and Tukey's post hoc test.

Based on previous reports [18] and our findings indicating that MB promotes lipid peroxidation and GSH depletion (Figures 3(d) and 8(b)), we hypothesized that ferroptosis might be involved in the cellular fate. Ferroptosis is a new discovered non-apoptotic-dependent cell death characterized by lipid peroxidation and GSH depletion [31], which can be counteracted by the ferroptotic inhibitor Fer-1. As ferroptosis is associated with the downregulation of glutathione peroxidase 4 (GPX4) [32], we evaluated mRNA expression in our model. We found that in cells with endogenous *α*-synuclein expression, MB exposure decreased GPX4 expression (Figure 9(a)) together with a rise in lipid peroxidation and GSH reduction (Figures 3(d) and 8(b)) and the absence of apoptotic nucleus (data not shown), being all ferroptosis markers. In cells with *α*-synuclein overexpression treated with MB, no changes in GPX4 levels or GSH content were detected (Figures 9(a) and 8(b)). To further study the effect of *α*-synuclein overexpression on MB-mediated ferroptosis, Fer-1 was used. As shown in Figure 9(b), the inhibition of ferroptosis reduced ROS production to a larger extent in control cells in the presence of the pesticide, thus suggesting that the contribution of ferroptosis was ameliorated by *α*-synuclein overexpression. To reinforce these data, the MitoTracker Red probe was used as mitochondrion specific dye to provide insights into mitochondrial alterations under ferroptosis inhibition. We found that the dye was localized into mitochondria in control neurons, whereas it was diffused throughout the cytoplasm and nucleus in control cells treated with MB, as shown in Figure 9(c). This effect was proposed as a mitochondrial membrane potential qualitative defect [26]. However, in the presence of Fer-1, the mitochondrial profile resembled control cells. The same effect was observed when fluorescence was quantified (RawIntDen), being higher MitoTracker intensity in MB-exposed control cells than in the rest of the experimental conditions. Regarding cells overexpressing *α*-synuclein, no changes were detected upon MB exposure (Figure 9(c)). Altogether, our results suggest that middle *α*-synuclein overexpression prevents MB-induced ferroptosis.

## 4. Discussion

Increasing body of evidence from epidemiological studies has demonstrated pesticide exposure as a risk factor for PD [5, 6]. Studies performed in MB- and paraquat-treated mice demonstrated decreased tyrosine hydroxylase expression and altered dopamine metabolism in the *substantia nigra*, accompanied by impaired motor activity [7, 8]. It has also been reported that MB exposure is able to upregulate *α*-synuclein expression in neuronal and epidermal cell lines [4]. It is well-known that *α*-synuclein overexpression, accumulation, and aggregation are considered PD hallmarks [1, 2, 33].

Our laboratory has previously demonstrated that the overexpression of the wild-type *α*-synuclein form or its mutated variant, A53T, does not alter cell viability but triggers a metabolic switch in neurons [34, 35]. Furthermore, we have shown that *α*-synuclein overexpression promotes changes in neuronal cytoskeleton and lipid accumulation. Indeed, both events are related to the loss of neuronal markers and function but, at the same time, necessary for conferring neuroprotective strategies against different types of injury, such as oxidative stress and proteostasis impairment [24, 34, 35]. In line with this, neurons overexpressing A53T *α*-synuclein display lower ROS and lipid peroxidation levels when exposed to iron-induced oxidative stress [34]. Moreover, our lab has demonstrated that the characteristic lipid accumulation of wt *α*-syn neurons is part of a neuroprotective response elicited by insults, such as proteasomal inhibition and manganese toxicity [35].

Taking into account our previous findings and the fact that the interaction between MB exposure and *α*-synuclein biology has not been deeply investigated, our aim was to characterize neuronal signaling events related to antioxidant defenses in neurons with endogenous levels and middle overexpression of *α*-synuclein. Specifically, we focused our interest on FOXO3a and NRF2 transcription factors, both involved in cellular responses associated to oxidative stress.

MB exposure was able to induce cell death and the increase in oxidant levels in cells with endogenous *α*-synuclein expression. In line with our previous findings, cells with overexpression of *α*-synuclein were less vulnerable to MB-induced oxidative stress and death. To dissect the mechanisms involved in this neuroprotective effect and taking into account that MB exposure triggers oxidative stress, we first evaluated NRF2-regulated signaling. This transcription factor belongs to Cap'n'Collar family of the basic leucine zipper proteins [36]. It is a key regulator for the antioxidant response element (ARE) promoter region, regulating the expression of antioxidant and phase-II detoxifying enzymes such as GCL and HO-1 [37]. Control and wt *α*-syn cells displayed a similar NRF2 nuclear localization pattern that was increased to the same extent when exposed to MB. However, whereas wt *α*-syn cells showed unaltered mRNA levels of *GCLc*, the expression of this NRF2 downstream gene was dramatically decreased in control cells when exposed to MB. A similar effect was observed for *HO-1* mRNA levels. These results demonstrate that MB exposure has a different impact on the expression of NRF2-dependent antioxidant defenses related to the extent of *α*-synuclein expression. Once NRF2 is located in the nucleus, a decrease in the inhibitory BACH1 protein is required for its binding to DNA at the ARE promoter region [38]. Since NRF2 nuclear localization was similar in control and wt *α*-syn cells exposed to the pesticide, and to discern the mechanism involved in *GCLc* and *HO-1* expression, we next explored the state of the transcription factor repressor BACH1. After MB treatment, the expression of BACH1 was increased in controls, whereas nuclear BACH1 localization remained unaltered in cells overexpressing *α*-synuclein. These results suggest that NRF2 downregulation after MB exposure occurs through an increase in the repressor BACH1 in control cells. This scenario is different in neurons overexpressing *α*-synuclein since BACH1 expression remains unaltered even after MB treatment. A similar mechanism was reported in Zn-deficient IMR-32 cells challenged with dopamine [25].

Another crucial transcription factor related to antioxidant defenses in neurons is FOXO3a [39]. We have previously demonstrated that FOXO3a is modulated by PI3K/AKT pathway to promote neuroprotection against oxidative stress and oligomeric *β*-amyloid peptide in hippocampal neurons [12, 13]. Here, we show that nuclear localization of the nonphosphorylated active form of FOXO3a in neurons overexpressing *α*-synuclein is higher than in control cells. In addition, when challenged with MB, FOXO3a nuclear localization is higher in wt *α*-syn cells. We confirmed that, in connection with the higher nuclear localization of the active form of FOXO3a observed in wt *α*-syn cells, these cells presented higher levels of SOD2 expression and catalase activation upon MB treatment. These results support the protective effect against MB neurotoxicity observed in cells overexpressing *α*-synuclein.

Subcellular localization and posttranslational modifications are involved in the modulation of FOXO3a transcriptional activity [40]. One of the most described mechanisms for negatively regulating FOXO3a transcriptional activity is its phosphorylation by PI3K/AKT pathway. Once phosphorylated by AKT, the inactive FOXO3a form is unable to bind to DNA and finally exported to the cytosol [12, 13, 40]. Surprisingly, phosphorylated FOXO3a levels were not altered by MB exposure and were similar in control and wt *α*-syn cells. The pharmacological inhibition of PI3K allows us to confirm that the regulation of FOXO3a during MB toxicity is independent of the phosphorylation mediated by PI3K/AKT pathway. Another described posttranslational modification that regulates FOXO3a activity is acetylation/deacetylation [41, 42]. In this connection, the deacetylase SIRT1 promotes antioxidant responses by FOXO3a deacetylation [42]. Specifically, SIRT-1 confers neuroprotection against oxidative stress by regulating SOD2 and catalase expression and activities [30, 43]. We found that *α*-synuclein overexpression was related to an increase in the deacetylase SIRT1 expression. In addition, TSA was able to induce an increase in oxidative stress markers in control cells exposed to MB, this effect being attenuated by *α*-synuclein overexpression. These results indicate that SIRT1 is part of the neuroprotective strategy against MB toxicity in *α*-synuclein overexpressing cells.

Ferroptosis is a form of cell death triggered by the failure of antioxidant defenses leading to oxidative stress with lipid peroxidation and GSH depletion [31]. MB has been proposed as a triggering factor of ferroptosis in dopaminergic neurons [18], which is supported by our present work. MB-exposed cells with *α*-synuclein endogenous expression exhibited the presence of ferroptosis markers, such as upregulated lipid peroxidation, reduction of GSH content, and GPX4 expression. Paradoxically, *α*-synuclein overexpression prevented GSH and GPX4 downregulation in cells exposed to the pesticide, which were less sensitive to the ferroptosis inhibitor Fer-1. Previous findings have demonstrated that aggregated *α*-synuclein promotes ROS production and lipid peroxidation within the membrane, thus increasing the interactions between oligomers and membranes and consequently inducing calcium influx and finally ferroptosis [44]. Our scenario might represent a previous stage of this injury mediated by *α*-synuclein aggregation.

**Figure 10:**
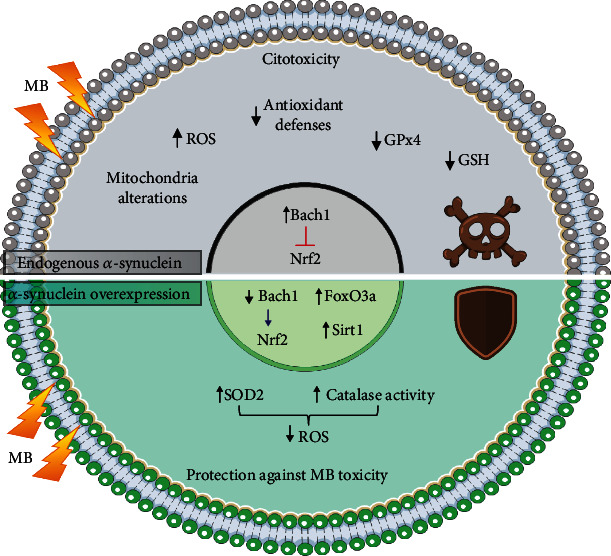
Concluding remarks. Mechanisms of neuroprotection against MB injury triggered by middle *α*-synuclein overexpression involving antioxidant response and ferroptosis. The figure was partly generated using Servier Medical Art, provided by Servier, licensed under a Creative Commons Attribution 3.0 unported license.

In summary, our results show that middle overexpression of *α*-synuclein (200% higher than endogenous levels) attenuates MB-induced injury through a concerted antioxidant response that involves the modulation of NRF2, FOXO3a, and SIRT1. The fact that MB is able to induce the increase of *α*-synuclein expression in control cells argues in favor of a cytoprotective role. This allows us to hypothesize that under middle overexpression of *α*-synuclein, resembling early stages of PD, several mechanisms of neuroprotection are activated (Figure 10). However, it can not be discarded that probably these mechanisms become overwhelmed in scenarios of persistent injury (chronic exposure of toxicants or high proteostasis impairment), tipping the balance towards neuronal damage and death.

## Data Availability

Data is available within the article and on request from the authors.
